# Marine vampires: Persistent, internal associations between bacteria and blood-feeding marine annelids and crustaceans

**DOI:** 10.3389/fmicb.2022.1113237

**Published:** 2023-01-11

**Authors:** Shana K. Goffredi, Ralph G. Appy, Rebecca Hildreth, Julia deRogatis

**Affiliations:** ^1^Department of Biology, Occidental College, Los Angeles, CA, United States; ^2^Cabrillo Marine Aquarium, San Pedro, CA, United States

**Keywords:** obligate blood-feeding, hematophagous, symbiosis, marine leech, parasitic isopod, parasitic copepod, *Vibrio*

## Abstract

Persistent bacterial presence is believed to play an important role in host adaptation to specific niches that would otherwise be unavailable, including the exclusive consumption of blood by invertebrate parasites. Nearly all blood-feeding animals examined so far host internal bacterial symbionts that aid in some essential aspect of their nutrition. Obligate blood-feeding (OBF) invertebrates exist in the oceans, yet symbiotic associations between them and beneficial bacteria have not yet been explored. This study describes the microbiome of 6 phylogenetically-diverse species of marine obligate blood-feeders, including leeches (both fish and elasmobranch specialists; e.g., *Pterobdella, Ostreobdella,* and *Branchellion*), isopods (e.g., *Elthusa* and *Nerocila*), and a copepod (e.g., *Lernanthropus*). Amplicon sequencing analysis revealed the blood-feeding invertebrate microbiomes to be low in diversity, compared to host fish skin surfaces, seawater, and non-blood-feeding relatives, and dominated by only a few bacterial genera, including *Vibrio* (100% prevalence and comprising 39%–81% of the average total recovered 16S rRNA gene sequences per OBF taxa). *Vibrio* cells were localized to the digestive lumen in and among the blood meal for all taxa examined *via* fluorescence microscopy. For *Elthusa* and *Branchellion, Vibrio* cells also appeared intracellularly within possible hemocytes, suggesting an interaction with the immune system. Additionally, *Vibrio* cultivated from four of the obligate blood-feeding marine taxa matched the dominant amplicons recovered, and all but one was able to effectively lyse vertebrate blood cells. Bacteria from 2 additional phyla and 3 families were also regularly recovered, albeit in much lower abundances, including members of the Oceanospirillaceae, Flavobacteriacea, Porticoccaceae, and unidentified members of the gamma-and betaproteobacteria, depending on the invertebrate host. For the leech *Pterobdella*, the Oceanospirillaceae were also detected in the esophageal diverticula. For two crustacean taxa, *Elthusa* and *Lernanthropus*, the microbial communities associated with brooded eggs were very similar to the adults, indicating possible direct transmission. Virtually nothing is known about the influence of internal bacteria on the success of marine blood-feeders, but this evidence suggests their regular presence in marine parasites from several prominent groups.

## Introduction

Hematophagy, or blood feeding, has emerged as an extremely successful nutritional strategy in thousands of species of animals from across the animal tree of life (Husnik, 2018). Considerable efforts have been made to understand the importance of these parasites in host vertebrate population dynamics, competition, energy flow and biodiversity (Holmes, 1996; Hudson et al., 2006; Wood et al., 2007). As a group, blood-feeding parasites are important influences on food webs and ecosystem health by both directly affecting the fitness and abundance of their immediate host, and also aiding the survival of predators of those species by increasing prey susceptibility to predation (Dunne et al., 2013). Obligate blood-feeding (OBF) invertebrates display a diversity of adaptations to accommodate blood feeding, including modifications of behavior, morphology, biochemistry and microbiology. Behaviorally, they locate and attach to their host prey using specific sensory mechanisms and questing-like behavior (Chaisson and Hallem, 2012). Morphologically, they often possess hooks and suckers, and highly extensible digestive systems, as well as sturdy body walls, to withstand extreme expansion during blood consumption (Khan, 1982; Sawyer, 1986; Graf, 2002). Because of this, OBF species can take large blood meals, many times their body weight, feed infrequently (e.g., every 6–12 months), and rely on comparably long digestion times (e.g., up to 14 days; Zebe et al., 1986; Lane, 1991). Biochemically, they produce vasodilators, anesthetics and anticoagulants to relieve venous constriction and ensure blood flow from their vertebrate hosts (Ribeiro, 1987). Finally, OBF taxa must contend with vitamin deficiencies (especially B vitamins), red blood cells that are difficult to digest, and heme toxicity (Toh et al., 2010). To overcome these dietary hurdles, they partner with internal bacteria that are believed to play an important role in counteracting the low digestibility and vitamin B deficiency, specifically.

Beneficial attributes of internal bacteria are generally wide ranging, from protection from predation or abiotic stresses to the dietary provision of missing nutrients or breakdown of foodstuffs. The possibility that internal bacteria could compensate for an unbalanced and difficult-to-digest blood meal was initially supported by research in the mid-1900s on medicinal leeches (see Jennings and Van Der Lande, 1967 and references therein). For terrestrial OBF species (i.e., tsetse fly, vampire bat), an alliance with bacteria appears to be necessary for animals to occupy the unusual niche of solely relying on blood for nutrition. Many studies since have provided evidence for high mortality rates of OBF species when deprived of their gut microbes (Lake and Friend, 1968; Rio et al., 2016), indicating a symbiotic relationship with bacteria as key to their success. Although experimental evidence is still limited, provisioning of B vitamins by bacterial symbionts has been demonstrated for blood-feeding arthropods, including tsetse flies and ticks (Sang, 1956; Pais et al., 2008). This has been investigated using genomic techniques, with evidence that bacterial symbionts found in the midgut of tsetse flies not only compensate for nutritional deficiencies of the invertebrate host, but also possibly complement other co-occurring resident bacteria (Akman et al., 2002; Toh et al., 2006; Snyder et al., 2010). In this way, persistent bacterial presence is believed to play an important role in parasite adaptation to blood-feeding, a specific niche that is generally unavailable to most animals.

Numerous OBF invertebrates also thrive in the marine realm, parasitizing bony and cartilaginous fish in the worlds’ oceans. These include leeches, crustaceans, nematodes, and flatworms, to name a few. Like their terrestrial relatives, marine OBF species negatively influence their hosts, causing anemia, blindness, decreased reproductive fitness, and mortality, especially in bony fish populations, depending on the age of the fish and the number of infecting parasites (Kabata, 1981; Khan, 2012). For marine OBF species, an alliance with bacteria is also expected, but has so far not been well studied. For example, the Piscicolidae is a large family of marine leeches comprising ~60 genera and 200 species, yet only a single study documents a possible relationship with internal bacteria (Goffredi et al., 2012). This group of marine leeches is a likely candidate for the most ancestral clade within the annelid subclass Hirudinea (Utevsky et al., 2007), thus the investigation of this family is generally important for determining whether bacterial-mediated digestion and provisioning of complementary nutrients might be a key innovation in all blood-feeding leeches. Additionally, marine isopods within the family Cymothoidae include numerous species with a unique parasitic lifestyle—the exclusive consumption of vertebrate blood from the gills of primarily bony fish (Brusca, 1978, 1981). So far there have been very few studies related to the feeding biology of cymothoid isopods, from the perspectives of functional morphology of mouthparts or possible bacterial symbioses (Nagler and Haug, 2016). As with the piscicolids, there is a single publication on the possible relationship between bacteria and a species of blood-feeding Antarctic isopod (*Gnathia calva*; Juilfs and Wägele, 1987). Finally, the large and diverse copepod order Siphonostomatoida (> ~1,000 species) parasitize shallow- and deep-water fish worldwide, including farmed salmon and cod (Perkins, 1983). These parasites can consume so much blood that they negatively affect fish fitness, competition, and reproduction (Kabata, 1979; Godwin et al., 2015), thereby impacting not only natural ecosystems, but fish stock production for human consumption. Despite their omnipresence in the ocean, siphonostomatid copepods remain entirely unexplored with regard to symbiotic partnerships.

**Figure 1:**
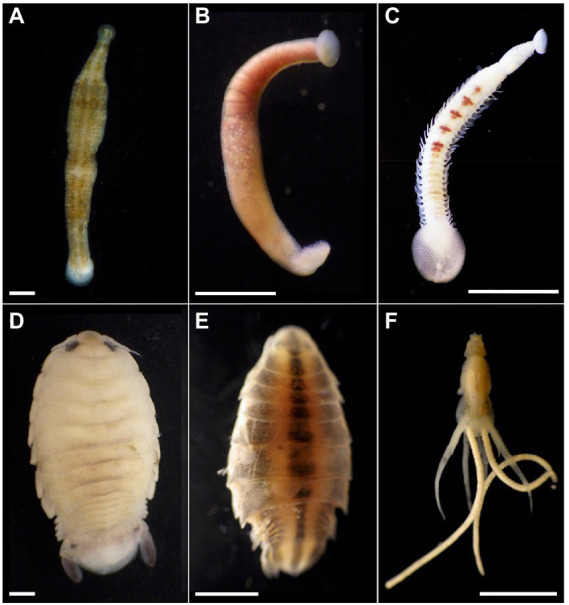
Marine blood-feeding invertebrates examined in this study. The bony fish leeches **(A)** *Pterobdella occidentalis* and **(B)** *Ostreobdella californiana*. Scale bars 1 mm. **(C)** The elasmobranch leech *Branchellion lobata.* Scale bar 5 mm. **(D)** The isopods *Elthusa vulgaris* and **(E)** *Nerocila californica*. Scale bars 2 mm. **(F)** The copepod *Lernanthropus latis*. Scale bar 4 mm. Photo credits: S. Goffredi.

Possible alliances between beneficial bacteria and obligate blood-feeding marine invertebrates were examined for two dominant categories of known blood-feeding marine invertebrates (leeches and crustaceans), collected from southern California coastal waters. Using DNA sequencing analysis combined with fluorescence microscopy, and bacterial cultivation, we document the prevalence of bacteria in 6 marine OBF species; fish and shark leeches (e.g., *Pterobdella, Ostreobdella*, and *Branchellion*), the isopods *Elthusa* and *Nerocila*, and the copepod *Lernanthropus* (Figure 1). Blood-feeding animals are not only important to study because of their potential symbiotic relationships with microbes, but because of their ability to act as both vectors for pathogens and the harm they cause to fish stocks. Surveying the microbial communities associated with marine blood-feeding invertebrates not only increases knowledge about nested biological diversity in the ocean, but may also provide insight into their successful nutritional strategy of parasitizing marine vertebrates.

## Materials and methods

### Sample collection

A variety of fish and elasmobranchs were collected from the coastal waters of southern California, either by hand or *via* trawl onboard expeditions in collaboration with the Orange County Sanitation District (OCSD)—Environmental Laboratory and Ocean Monitoring team. In all cases, permits to collect the fish hosts, from which we removed invertebrate parasites and, in most cases released, were held by R.A. (SC-13105), S.G. (SC-10578), and S-190710005-22077-001. The leech *Pterobdella occidentalis* (Goffredi et al., in press) was collected *via* minnow traps mainly from longjaw mudsuckers (*Gillichthys mirabilis*), plus a few additional host fish (Supplementary Table S1), while *Branchellion lobata* Moore 1952 was collected from various ray species, by beach seine, including primarily the Pacific round ray (*Urobatis halleri*) and bat rays (*Myliobatis californica*) in trawls off of Los Angeles or in San Diego Bay. The leech *Ostreobdella californiana* Burreson et al., 2019 was provided by Freeland Dunker, a veterinarian at the California Academy of Sciences. The species was originally described from rockfishes in the genus *Sebastes* in public display tanks in the Steinhart Aquarium, California Academy of Sciences, San Francisco, CA, although it has also been observed in nature on *Sebastes* from the San Francisco and Monterey Bay areas (Burreson et al., 2019). Marine isopods within the family Cymothoidae, including *Elthusa vulgaris* Stimpson, 1857 and *Nerocila californica* Schioedte and Meinert, 1881 were collected from various bony fish hosts, including Pacific sanddabs (*Citharichthys sordidus*) or killifish (*Fundulus parvipinnis*), by either beach seine or trawls. In some cases, the host fish was unknown, since cymothoids often detach from their host fish in trawls or seines (Brusca, 1978). The marine copepod *Lernanthropus latis* Yamaguti, 1954 (order Siphonostomatoida) was collected primarily from California corbina (*Menticirrhus undulatus*) by hook and line. Additionally, eggs were taken from *Elthusa* marsupium and egg masses were excised from adult *Lernanthropus* (*n* = 3 each). *Pterobdella* cocoons (*n* = 3 pooled collections) were recovered from surfaces of the collection vials, while in captivity. Specimens for molecular analysis were preserved within 2 h of collection in ~90% ethanol and stored at 4°C.

Non-blood feeding crustaceans, including sea lice within the Caligidae (*Trebius* and *Lepeophtheirus*), were collected from the external surfaces of Pacific Round rays (*U. halleri*) and the California skate (*Raja inornata*), with *Clausidium vancouverensis* removed from the ghost shrimp (*Neotrypaea californiensis*). Ghost shrimp exoskeletons were also analyzed, as were swabs of skin mucus collected *via* sterile cotton swab from host fish. Swabs were stored at –80°C prior to molecular analysis. Seawater samples were taken from three collection locations and filtered (2 l) onto a 0.22 μm Sterivex-GP polyethersulfone filter (Millipore-Sigma, St. Louis, MO, United States) and stored at -80°C until DNA analysis.

### Bacterial cultivation

Bacteria from the digestive systems of living specimens of *Branchellion*, *Pterobdella*, *Elthusa* and *Lernanthropus* were cultivated by plating homogenate (in 3X phosphate buffered saline using a ground glass tissue homogenizer) on Difco-BD Marine Agar 2,216. Plates were incubated at 25°C for 1–3 days under aerobic conditions, and single colonies were further purified by standard T-streak. Cells were suspended in alkaline PEG (60 g of PEG 200 with 0.93 ml of 2 M KOH and 39 ml of water). This suspension was then heated to 96°C for 20 min in order to lyse the bacterial cells and liberate the DNA. 16S rRNA gene was then amplified using the general primers 27F and 1492R (Lane, 1991), with an anneal temperature of 54°C. Successful 16S rRNA gene products were sequenced at Laragen, Inc. (Culver City, CA). Hemolysis activity was assessed qualitatively *via* zone of lysis on blood agar plates (10% blood in Tryptic Soy Agar; Supplementary Figure S1). Bacteria capable of hemolysis created an obvious zone of depletion, at 25°C. Individual cell morphology was determined *via* scanning electron microscopy (SEM; Supplementary Figure S1). Bacterial cells for SEM were initially fixed in 3% glutaraldehyde in 0.1 mol L-1 cacodylate for 72 h at 4°C. Samples were then pulled onto a 0.22 μm polycarbonate filter (Millipore, Billerica, MA), washed in a graded ethanol series (50%, 75%, and 100%) and placed in hexamethyldisilazane for 1 h at room temperature. Filters were then mounted, palladium-coated (Hummer VI, Union City, CA), and visualized using a Phenom desktop SEM (FEI Instruments, Hillsboro, OR).

### DNA extraction and 16S rRNA gene sequencing

Total genomic DNA was extracted from specimens, that had been rinsed in ethanol and dried, using the Qiagen DNeasy kit (Qiagen, Valencia, CA, United States) according to the manufacturer’s instructions. Swab and filter extractions deviated from manufacturer’s instructions by doubling reagent volumes during the initial lysis and incubation step (i.e., 360 μl ATL buffer and 40 μl Proteinase K) to cover the entire swab or filter area. The V4-V5 region of the 16S rRNA gene was amplified using bacterial primers with Illumina (San Diego, CA, United States) adapters on the 5′ end 515F (5′-TCGTCGGCAGCGTCAGA-TGTGTATAAGAGACAGGTGCCAGCMGCCGCGGTAA-3′) and 806R (5′-GTCTCGTG-GGCTCGGAGATGTGTATAAGAGACAGGGACTACHVGGGTWTCTAAT-3′; Caporaso et al., 2011). Note: Eight swabs and one non-blood-feeding isopod were not amplifiable for the 16S rRNA gene. The PCR reaction mix was set up in duplicate for each sample with Q5 Hot Start High-Fidelity 2x Master Mix (New England Biolabs, Ipswich, MA, United States) and annealing conditions of 54°C for 25 cycles. Duplicate PCR samples were then pooled, and 2.5 μl of each product was barcoded with Illumina NexteraXT index 2 Primers that include unique 8-bp barcodes (P5 5′-AATGATACGGCGACCACCGAGATCTACAC-XXXXXXXX-TCGTCG GCAGCGTC-3′ and P7 5′-CAAGCAGAAGACGGCATACGAGAT-XXXXXXXX-GTCTCGTGGGCTCGG-3′). Secondary amplification with barcoded primers used conditions of 66°C annealing temperature and 10 cycles. Products were purified using Millipore-Sigma (St. Louis, MO, United States) MultiScreen Plate MSNU03010 with a vacuum manifold and quantified using Thermo Fisher Scientific (Waltham, MA, United States) QuantIT PicoGreen dsDNA Assay Kit on a BioRad CFX96 Touch Real-Time PCR Detection System. Barcoded samples were combined in equimolar amounts into a single tube and purified with Qiagen PCR Purification Kit 28,104 before submission to Laragen (Culver City, CA, United States) for 2 × 250 bp paired end analysis on the Illumina MiSeq platform with PhiX addition of 20%.

Amplicon sequence data was processed in Quantitative Insights Into Microbial Ecology (v1.8.0). Raw sequence pairs were joined and quality-trimmed using the default parameters in QIIME. Sequences were clustered with 99% similarity using the UCLUST open reference clustering protocol, and then, the most abundant sequence was chosen as a representative for each. Taxonomic identification for each representative sequence was assigned using the Silva-138 database, and checked *via* BLAST. Quantification and statistical analyses are described in the Results sections and figure legends. Comparisons were performed using ANOVA and statistical significance was declared at *p* < 0.05. Non-metric multidimensional scaling ordination (NMDS), analysis of similarity (ANOSIM), and similarity percentage analysis (SIMPER) analyses were performed in Primer-E, after square-root transforming the dataset and calculating Bray–Curtis similarities (Clarke and Warwick, 2001). The raw Illumina 16S rRNA gene barcode sequences and metadata collected in this study are available from the NCBI Small Read Archive (BioProject # PRJNA910167). The processed sequence data, as well as representative sequences, are available on the Dryad Digital Repository URL https://doi.org/10.5061/dryad.vmcvdncx9.

For at least one specimen from each OBF taxon, a 16S rRNA gene clone library was generated using the general primers 27F and 1492R (Lane, 1991) and the TOPO-TA kit (ThermoFisher, Waltham, MA, United States), according to the manufacturer’s instructions. In this way, longer sequences of the 16S rRNA gene were recovered for nearly all dominant bacterial ribotypes (*via* Laragen, Inc.; Supplementary Figure S1). Longer sequences were assembled using Sequencher v4.10.1 (GeneCodes Corp., Ann Arbor, MI, United States) and trees were generated using Geneious Prime v2022.2.1 (Biomatters, Inc. San Diego, CA, United States). 16S rRNA sequences for clones and bacterial isolates are available from GenBank under accession numbers OP981048-OP981071.

### Fluorescence *in situ* hybridization microscopy

Specimens for fluorescence *in situ* hybridization (FISH) microscopy were initially preserved in 4% sucrose-buffered paraformaldehyde (PFA) and kept at 4°C for 24–48 h. These PFA-preserved specimens were rinsed with 2× PBS, transferred to 70% ethanol, and stored at −20°C. Tissues were dissected and embedded in Steedman’s wax (1 part cetyl alcohol: 9 parts polyethylene glycol (400) distearate, mixed at 60°C). An ethanol: wax gradient of 3:1, 2:1 and 1:1, and eventually 100% resin, was used to embed the samples (1 h each treatment). Embedded samples were sectioned at 3 μm thickness using a Leica RM2125 microtome and placed on Superfrost Plus slides. Sections were dewaxed in 100% ethanol rinses. As a reference, some sections were histologically examined *via* the Wright stain (2.5 min exposure) and visualized. To specifically target the associated OBF *Vibrio*, a probe that was an exact match was used (*Vibrio* GV; 5′-AGGCCACAACCTCCAAGTAG-3′; Giuliano et al., 1999), labeled with the fluorochrome Cy3 at both the 3′ and 5′ terminus. For universal detection of most bacterial 16S rRNA genes, we used the probe EUB338 (Amann et al., 1990). The full comprehensive EUB338 probe set was not employed since probes EUB338-II and EUB338-III target Planctomycetales and Verrucomicrobiales, respectively, and 16S rRNA genes from these specific bacteria were not recovered *via* barcoding efforts. A nonsense probe (*NonEub*; 5′-ACTCCTACGGGAGGCAGC-3′) was used as a negative control. Hybridization buffers and wash buffers were prepared according to Pernthaler and Pernthaler (2005). The samples were incubated in hybridization buffer containing 50 nM probe at 46°C for 4–8 h, followed by a 15 min wash at 48°C. Sections were counterstained with 4′6’-diamidino-2-phenylindole (DAPI, 5 mg/ml) for 1 min, rinsed and mounted in Citifluor. Tissues were examined by epifluorescence microscopy using either a Nikon E80i epifluorescence microscope with a Nikon DS-Qi1Mc high-sensitivity monochrome digital camera or a Zeiss Elyra microscope with an ANDOR-iXon EMCCD camera. No FISH microscopy was conducted on *Ostreobdella*, due to insufficient initial preservation of the specimens, or *Nerocila*, due to low sample sizes.

### Transmission electron microscopy

A single *Pterobdella* specimen for TEM microscopy was initially preserved in 3% glutaraldehyde buffered with 0.1 M phosphate and 0.3 M sucrose (pH 7.8). Samples were washed in 0.1 M sodium cacodylate with 24% sucrose and post-fixed with 1% OsO4 in 0.1 M sodium cacodylate for 1 h. Samples were stained in 3% uranyl acetate in 0.1 M sodium acetate buffer for 1 h, dehydrated in ethanol and embedded in Spurr’s resin. Thick sections (0.4 μm) were stained with methylene blue and examined using a Nikon E80i microscope, while thin sections (70 nm) were stained with lead citrate and examined using a Zeiss EM 109 transmission electron microscope.

## Results and discussion

### The microbiomes of obligate blood feeding marine invertebrates

**Figure 2:**
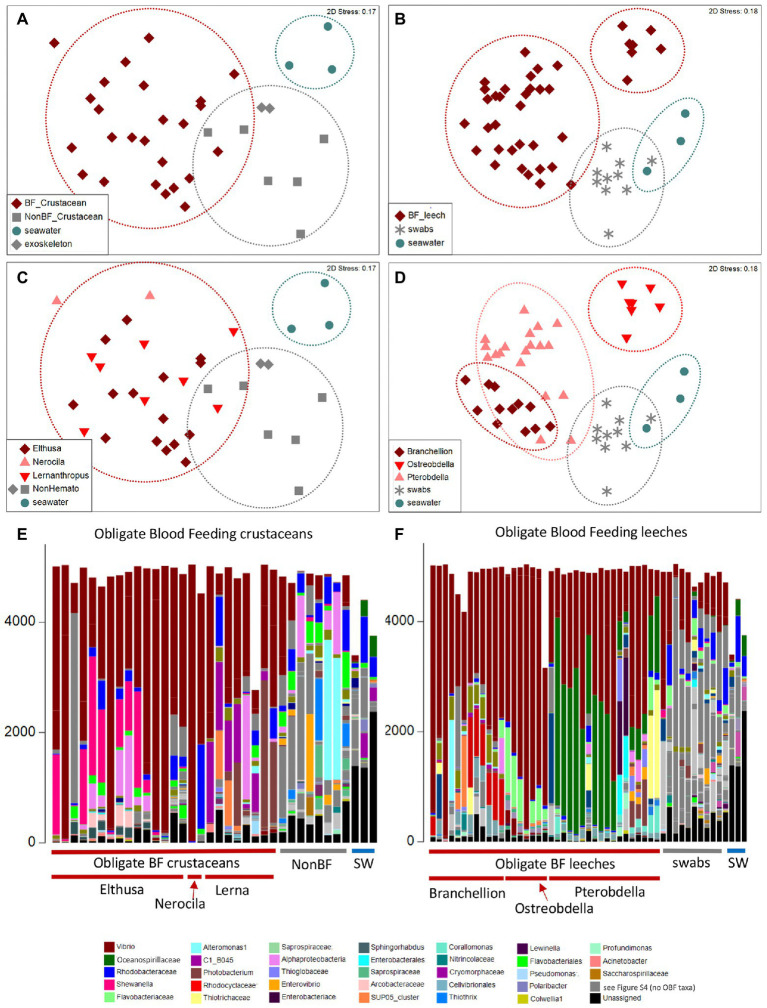
Microbiome diversity analysis of obligate blood feeding (OBF) crustaceans and leeches, based on 16S rRNA gene sequence similarity, using Non-metric multidimensional scaling (NMDS) ordination plots based on Bray–Curtis similarity resemblance, at the **(A,B)** broad category level, including blood-feeding versus comparison samples of non-blood feeding (NonBF) taxa, biological surfaces, and seawater (SW) and at the **(C,D)** specific blood-feeding taxa level. **(E,F)** Relative abundance of bacterial community structure at the genus level, from marine blood-feeders collected primarily from southern California coastal waters, including isopods *Elthusa* and *Nerocila*, the copepod *Lernanthropus* (Lerna), and leeches *Branchellion, Ostreobdella,* and *Pterobdella* (specific specimens are listed in Supplementary Table S1 in the same order as shown in the bar charts). Assigned bacterial taxa are color-coded as shown below. Taxa in dark gray were only found in the non-blood feeder (NBF) or seawater (SW) samples. Taxa in light gray were minor taxa in all specimens. See Supplementary Table S4 for a full key.

The microbiome was characterized for a number of diverse obligate blood-feeding marine invertebrate taxa, including the fish leeches *Pterobdella occidentalis* and *Ostreobdella californiana*, the elasmobranch leech *Branchellion lobata,* the copepod *Lernanthropus latis*, and two isopod species, *Elthusa vulgaris* and *Nerocila californica*, the latter of which might also feed on tissue, in addition to blood (Bunkley-Williams and Williams, 1998; Figure 1; Supplementary Table S1). Bacterial 16S rRNA gene amplicon barcoding revealed that the microbiome of the OBF invertebrates was significantly distinct from non-blood-feeding relatives (in the case of crustaceans; ANOSIM R = 0.54, *p* = 0.01), swabs of fish prey species (in the case of the leeches; ANOSIM R = 0.43, *p* = 0.01), and surrounding seawater (ANOSIM R > 0.65, *p* = 0.01 for both; Figure 2; Supplementary Table S2). Blood-feeding invertebrate species had a significantly lower microbiome diversity (average Shannon index for each species was 0.5–1.5, at the 99% similarity 16S rRNA gene level; Supplementary Table S1) compared to non-blood-feeding invertebrates, swabs of fish prey species, and surrounding seawater (average Shannon index of 1.9–2.5; ANOVA *p* < 0.0001). In past studies, comparatively lower microbial diversity has been observed for other OBF taxa, including the freshwater leech genera *Macrobdella* and *Hirudo* (Graf et al., 2006; McClure et al., 2021). One exception in this study was the copepod *Lernanthropus* with a slightly higher diversity (average Shannon index of 1.8 ± 0.4; Supplementary Table S1), comparable to the least diverse environmental sample. In all cases, the microbial community structure of each of the 6 obligate blood-feeding marine species was unique (ANOSIM R > 0.8, *p* = 0.01) and remained stable over 3–5 years of collection (Figure 2F; Supplementary Table S2), suggesting specific and non-transient associations.

**Figure 3:**
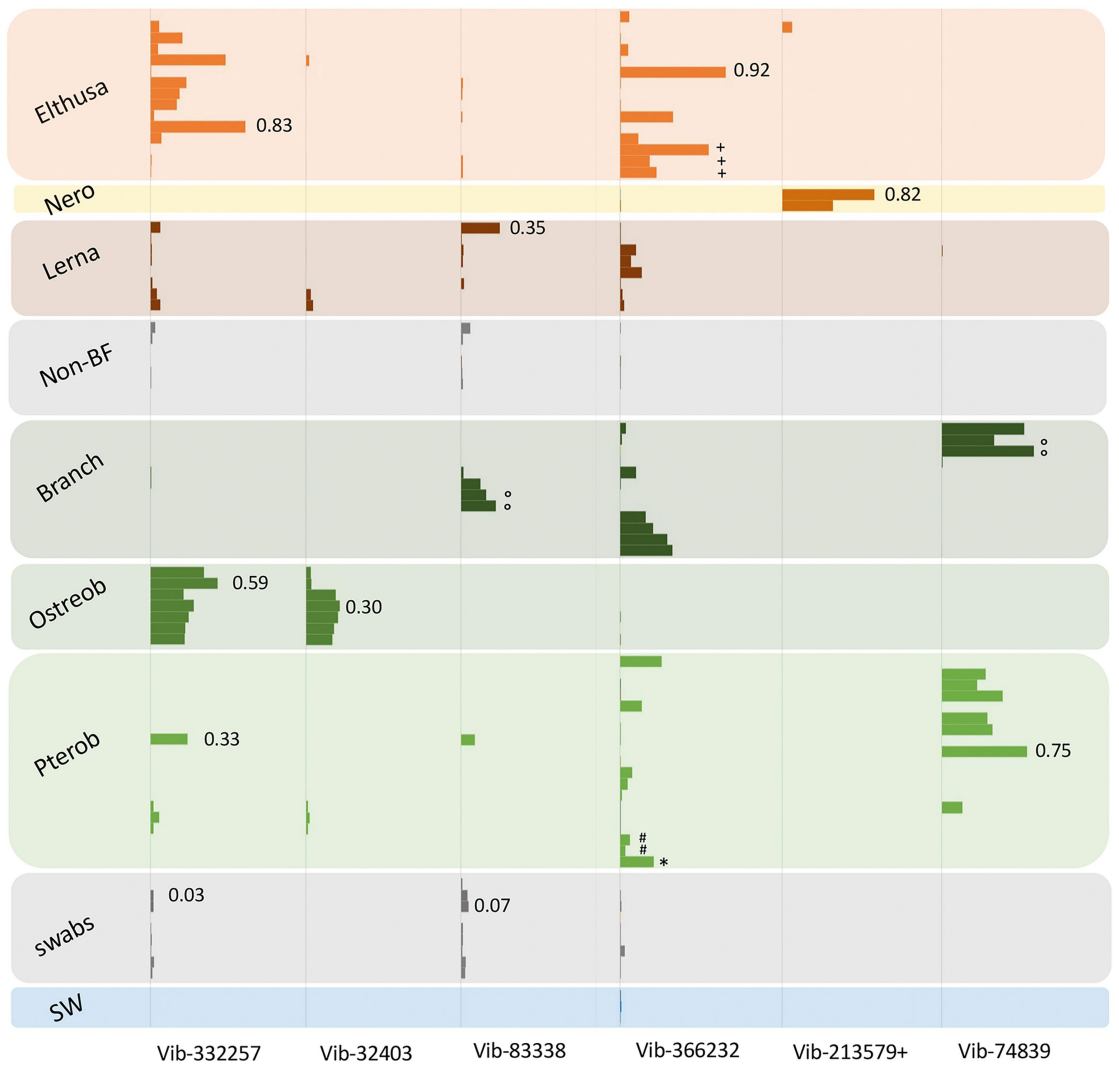
Relative abundances of the 7 most prevalent *Vibrio* ribotypes (based on 99% 16S rRNA gene sequence similarity) from obligate marine blood-feeders including the isopods *Elthusa* and *Nerocila* (Nero), copepod *Lernanthropus* (Lerna), and leeches *Branchellion, Ostreobdella,* and *Pterobdella,* compared to non-blood-feeding crustaceans (Non-BF), swabs of fish skin, and seawater (SW). Bars are scaled according to percent abundance of each *Vibrio* ribotype as a function of the entire microbial community in that specimen (for reference, occasional numbers indicate the % abundance of that bar). A total of 135 Vibrionaceae ribotypes were recovered from all samples in total, but the 7 portrayed here accounted for 80% of the total Vibrionaceae diversity. See Supplementary Figure S1 for the phylogenetic position of these ribotypes. Note the pooling of *Vibrio* ribotypes 19,850 with 213,579 (+) common only in the *Nerocila* specimens *via* amplicon sequencing. Occasional symbols note distinction within OBF taxa, including *Pterobdella occidentalis* recovered from the goby (#), and *P. abditovesiculata* from Hawaii (*), as well as *Branchellion* collected from rays other than the pacific ray (°), and *Elthusa* collected from killifish (+).

Blood-feeding invertebrate species were mostly dominated by only a few bacterial groups. In nearly all OBF species, ribotypes within the Vibrionaceae (*Vibrio*, *Alivibrio, Photobacterium*) were the most dominant member of the microbiome, comprising 39–81% of the total recovered 16S rRNA gene sequences, on average per OBF taxon (described in more detail below; Figure 2; Supplementary Figure S1; Supplementary Table S3). This was significantly higher than for non-blood feeding invertebrates and swab surfaces, both of which also hosted *Vibrio* (15%–18% of average recovered ribotypes per comparison group; ANOVA *p* = 0.0004 for OBF crustaceans versus non-blood feeders; ANOVA *p* = 0.00003 for OBF leeches versus fish swabs; Figures 2, 3; Supplementary Table S3). By contrast, seawater samples contained <2% *Vibrio*, based on recovered 16S rRNA genes (Figures 2, 3; Supplementary Table S3). The second most prominent associated bacterial ribotype depended on the specific OBF marine taxa, and included an unidentified member of the Flavobacteriaceae (22% on average recovered from *Ostreobdella*), an unidentified member of the Porticoccaceae (23% on average for *Lernanthropus*), *Shewanella* (21% on average for the *Elthusa*), and an unidentified member of the Oceanospirillaceae (34% average 16S rRNA gene sequences recovered from *Pterobdella*; Figure 2; Supplementary Table S3). For *Branchellion*, an undescribed gammaproteobacteria and betaproteobacteria co-occurred with *Vibrio*, each comprising ~15%–17% of recovered ribotypes (Figure 2; Supplementary Table S3). The maintenance of a gut community dominated by only 2–3 bacterial taxa has been observed for both *Macrobdella* and numerous hirudinid leeches (Graf et al., 2006; Kikuchi and Graf, 2007; Laufer et al., 2008; McClure et al., 2021). Many of the undescribed secondary bacterial ribotypes associated with the marine OBF taxa were dissimilar from known bacteria (< 90% 16S rRNA gene identity; Supplementary Figure S2), thus likely representing novel taxa. Some, however, including the Flavobacteriaceae and Porticoccaceae are known to associate with marine invertebrates (Garren et al., 2009; Webster et al., 2013; Dishaw et al., 2014), making them interesting candidates for follow-up studies on invertebrate endemic microbiome members. Within the marine OBF taxa, occasional bacteria and those present in much lower numbers, likely represent transient organisms, as observed in the medicinal leech (Worthen et al., 2006). For example, one *Nerocila* specimen revealed a near singular dominance of *Vibrio* (99% of ribotypes), whereas the other *Nerocila* specimen hosted both *Vibrio* and an unidentified member of the Rhodobacteraceae (22% of ribotypes), which was also found in appreciable numbers in *Elthusa*, non-blood feeding relatives, swabs, and seawater (>10% of ribotypes; Figure 2; Supplementary Table S3).

By comparison to the marine OBF taxa, very few bacterial ribotypes accounted for more than 10% of recovered sequences for the non-blood-feeding crustaceans, ghost shrimp exoskeletons, and swabs from fish skin (based on 99% 16S rRNA gene similarity). For those that did, some were shared with the OBF marine taxa, including *Pseudoalteromonas*, an undescribed alphaproteobacteria, and the aforementioned *Vibrio* and Rhodobacteraceae (Figure 2; Supplementary Table S3). Various other notable bacterial groups recovered from the environmental and non-blood-feeding samples were either not observed in the marine OBF taxa or were at very low levels, including *Alteromonas, Thiothrix, Rubitalea, Colwellia*, and *Marinomonas*, to name a few (Figure 2; Supplementary Table S3). This suggests a distinct, and winnowed, bacterial community associated with the marine OBF taxa compared to biological surfaces of invertebrates and vertebrates, non-blood feeding crustaceans, and seawater. This pattern is similar to previous reports of marine bacterial communities, which are distinctly reduced when associated with an animal host (Fraune and Bosch, 2007; Goffredi et al., 2021). Additionally, unassigned 16S rRNA gene sequences comprised 45% for the seawater samples and 9% for the non-blood-feeding taxa, compared to only 1–5% of the average 16S rRNA genes recovered from each marine OBF taxon (ANOVA *p* < 0.003).

Microbiome comparisons between lifestages differed for the various obligate blood feeding taxa. Eggs taken from the *Elthusa* marsupium and egg masses excised from adult *Lernanthropus* had very similar microbial communities to the adults, suggesting possible transmission of bacteria from parent to offspring (n = 3 each; ANOSIM R < 0.225, *p* > 0.14). Although additional fluorescent microscopy on the eggs would add additional evidence for vertical transmission, the similarities *via* 16SrRNA gene recovery included the community evenness of these specific bacterial taxa; *Vibrio* and *Shewenalla* in the case of *Elthusa* and *Vibrio* and Porticococcaceae for *Lernanthropus* (Supplementary Figure S3). The main difference from the adults was the significant occurrence of *Pseudoalteromonas* on the eggs of both species, which has been seen for other crustaceans (Gil-Turnes et al., 1989) and natural marine surfaces more broadly (Skovhus et al., 2007; Moisander et al., 2015). In crustaceans, the act of egg brooding has been shown to facilitate transfer of bacteria pseudovertically from parent to offspring (Goffredi et al., 2014; Sison-Mangus et al., 2015). *Pterobdella* cocoons, on the other hand, had very dissimilar microbiomes from the adults (n = 3 pooled collections; ANOSIM R = 0.98, *p* = 0.003), with comparatively high diversity values (avg H′ = 2.98, vs. 1.20 for the adults). Although cocoons could not be tracked to a specific parent, since they were found already deposited on surfaces, the microbial community demonstrated a specific reduction in *Vibrio*, a complete lack of the Oceanospirillaceae, and the presence of Rhodobacteraceae and numerous other groups not observed in the adults (Supplementary Figure S3). In leeches, some symbionts are thought to be transmitted vertically (Kikuchi and Fukatsu, 2002), for example in eggs that are carried by a brooding parent (Kikuchi and Fukatsu, 2002), while others, especially those in the crop that can be cultured in the lab, are suspected to be acquired from the environment, of either the cocoon or hatchlings.

### Consistent and distinct *Vibrio* associated with obligate marine blood feeders

As noted, bacteria within the *Vibrio* genus dominated the tissues of six phylogenetically-diverse OBF marine taxa, including leeches, isopods, and copepods. The most common *Vibrio* ribotypes, based on 16S rRNA gene sequencing, generally grouped into 4 *Vibrio* species clades, most closely related to *Vibrio tasmaniensis, V. parahaemolyticus/V. alginolyticus, V. anguillarum* and *V. atypicus*, previously recovered from environmental sources (Supplementary Figure S1). Out of a total of 135 distinct Vibrionaceae 16S rRNA gene amplicons recovered from all samples (based on 99% similarity), seven were dominant and accounted for 80% of the total Vibrionaceae diversity. These *Vibrio*-specific amplicons were either exclusive to the marine OBF specimens, or were present in much higher abundance, than the non-blood-feeding invertebrates and other nearby biological surfaces (Figure 3). For two of the blood-feeding taxa, identical *Vibrio* ribotypes were associated with specimens collected on different prey fish (e.g., *Pterobdella* from the longjaw mudsucker and goby, and *Elthusa* from killifish vs. Pacific sanddabs). Even the single specimen of *Pterobdella abditovesiculata* from the Hawaiian host fish *Eleotris* associated with the same *Vibrio* ribotype as those found in *Pterobdella occidentalis* from southern California.

Vibrionaceae species are well-known to associate, somewhat ubiquitously, with biological surfaces and plankton in the oceans (Montanari et al., 1999; Romalde et al., 2014). Although also known to degrade chitin and commonly associate with marine invertebrates, *Vibrio* species, in other studies, are mostly generalists and show little host preference (Preheim et al., 2011). However, some specific and persistent associations with beneficial Vibrionaceae have been detailed in other well-known systems, including the Hawaii bobtail squid and luminous fishes (Haygood and Distel, 1993; Visick et al., 2021). A specific association for the OBF marine taxa is suggested by evidence that the *Vibrio* ribotypes recovered were generally not associated with related invertebrates and nearby biological surfaces (Figure 3). Further, *Vibrio* were extremely abundant in the microbiome of *Elthusa, Branchellion* and *Nerocila* (e.g., comprising >96% of the total bacterial community in some individuals). By contrast, *Vibrio* species were detected, but low, in the microbiome of three temperate copepod species from the Gulf of Maine, with the most abundant Vibrionaceae genus comprising only 4% of all gamma-proteobacterial sequences recovered (Moisander et al., 2015). Finally, while pathogenic marine *Vibrio* species can be common, they are usually prevalent in only 20%–50% of hosts sampled at a given time (Davis and Sizemore, 1982; Sullivan and Neigel, 2018). The prevalence of *Vibrio* in the marine OBF taxa surveyed in this study was 100% and the pattern of *Vibrio* dominance and community structure continued for individuals collected over many years, thereby reducing the possibility of a transient bacterial infection.

**Figure 4:**
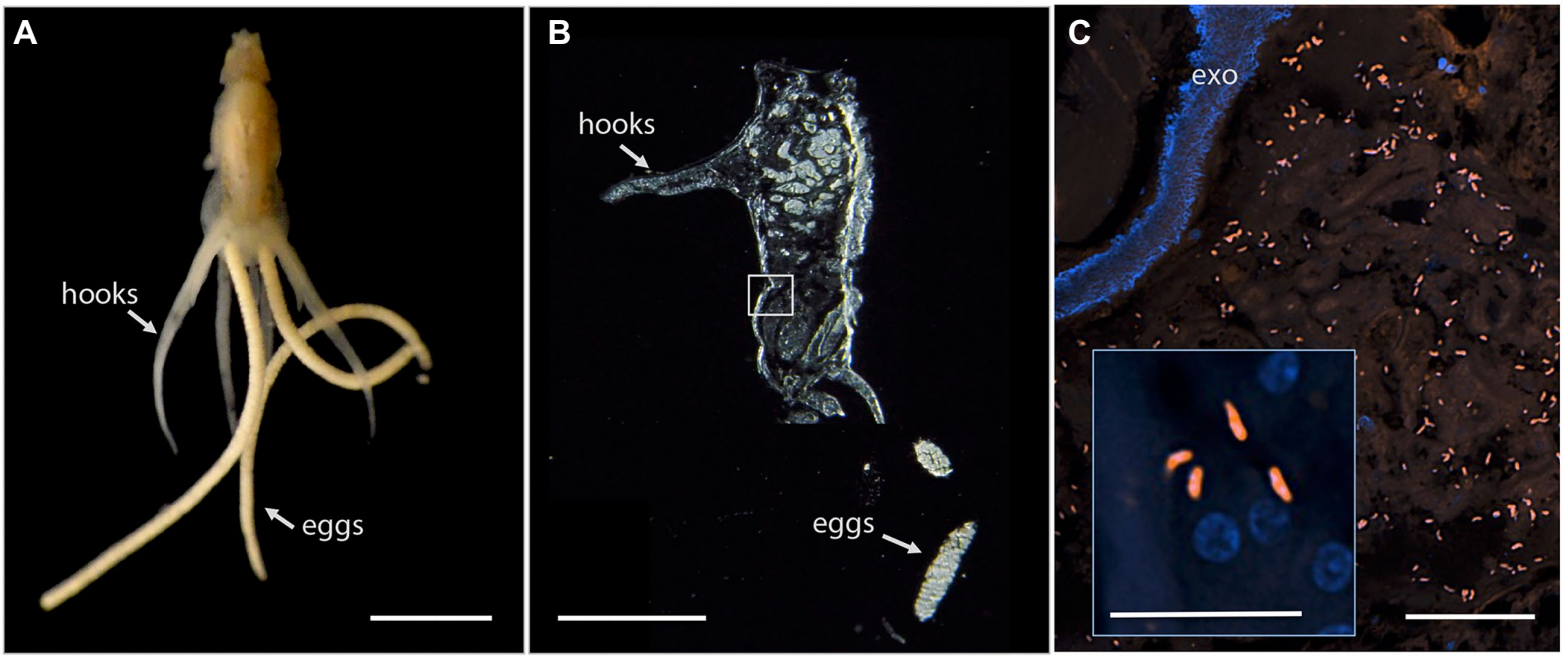
Fluorescent visualization and localization of *Vibrio* in **(A)** the parasitic copepod *Lernanthropus latis*, with clasping hooks and egg strings noted. Image taken with a Pentax WG-III handheld camera. Scale bar 2 mm. **(B)** Cross section of the specimen after being embedded in Steedman’s wax and sectioned. Scale bar 2 mm. **(C)** A *Vibrio*-specific fluorescent probe revealed comma-shaped bacterial cells, shown in orange *via* Cy3, within the digestive lumen space among bloodmeal. Scale bar 20 μm. (inset) A magnified view of the *Vibrio* cells, near copepod nuclei, shown in blue *via* DAPI. Scale bar 5 μm. exo, autofluorescent exoskeleton.

**Figure 5:**
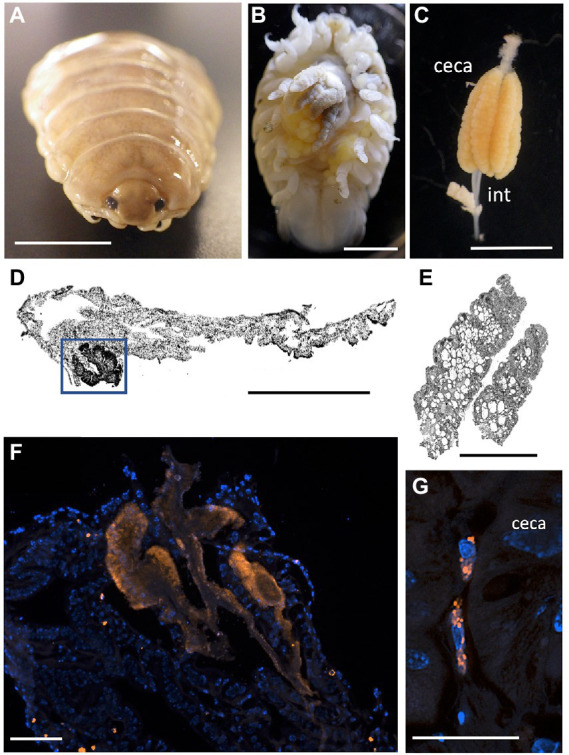
Fluorescent visualization and localization of *Vibrio* in **(A)** the parasitic isopod *Elthusa vulgaris*. **(B)** Ventral dissection showing the enlarged digestive ceca. Scale bar 5 mm. **(C)** Excised digestive ceca and intestine. Scale bar, 4 mm. A-C were taken with a Pentax WG-III handheld camera. **(D)** intestine sectioned and Wright stained. Scale bar, 1 mm. Square indicates region in **F**. **(E)** ceca sectioned and Wright stained. Scale bar, 1 mm. **(D,E)** Imaged *via* light microscopy. **(F)** A *Vibrio*-specific fluorescent probe, shown in orange with Cy3, revealed a strong signal within a darkly stained area of the intestine, near the ceca junction. Scale bar 100 μm. **(G)** *Vibrio* cells, shown in orange, observed inside of cells, at a specific junction between the midgut and the anterior digestive ceca. Scale bar 20 μm. Isopod nuclei are shown in blue, *via* DAPI. int., intestine.

**Figure 6:**
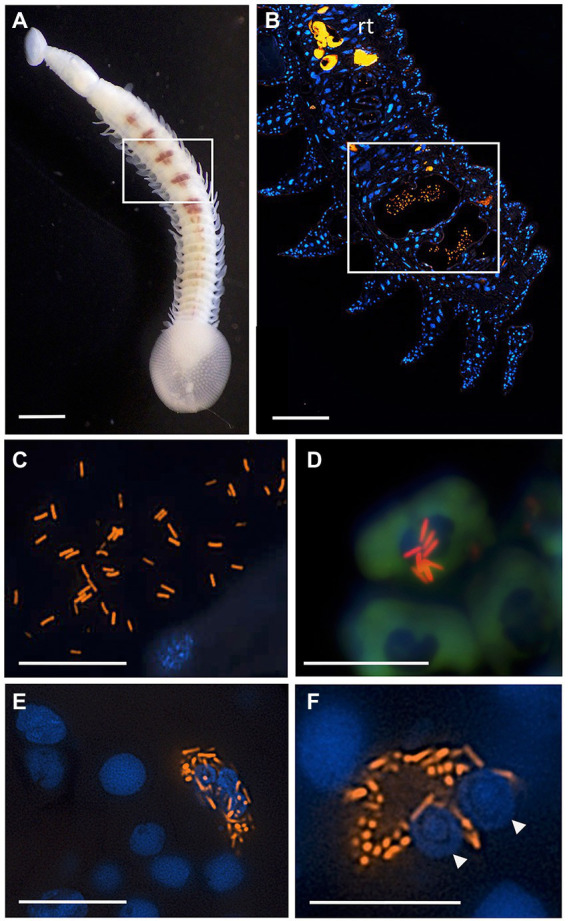
Fluorescent visualization and localization of *Vibrio* in **(A)** the elasmobranch leech *Branchellion lobata*. Square indicates regions of the crop filled with blood, as in **B**. Scale bar 1 mm. Taken with a Pentax WG-III handheld camera. **(B)** A partial longitudinal section through an individual, showing fluorescent hybridization using a *Vibrio*-specific probe to illuminate bacteria cells (shown in orange, *via* Cy3), with leech cell nuclei shown in blue (*via* DAPI). Square indicates region in **C–F**. Scale bar 100 μm. **(C,D)** A *Vibrio*-specific fluorescent probe revealed rod-shaped bacterial cells, shown in orange *via* Cy3, within the lumen space of the crop among bloodmeal (fish host blood cell shown in green, autofluorescence). **(D)** *Vibrio* cells appear to be on top of host cell and nucleus. **(E,F)** Within leech cells, sometimes in proximity to multiple nuclei (arrowheads), shown in blue *via* DAPI. **C-F** scale bars are 10 μm. rt., autofluorescent reproductive tissues.

**Figure 7:**
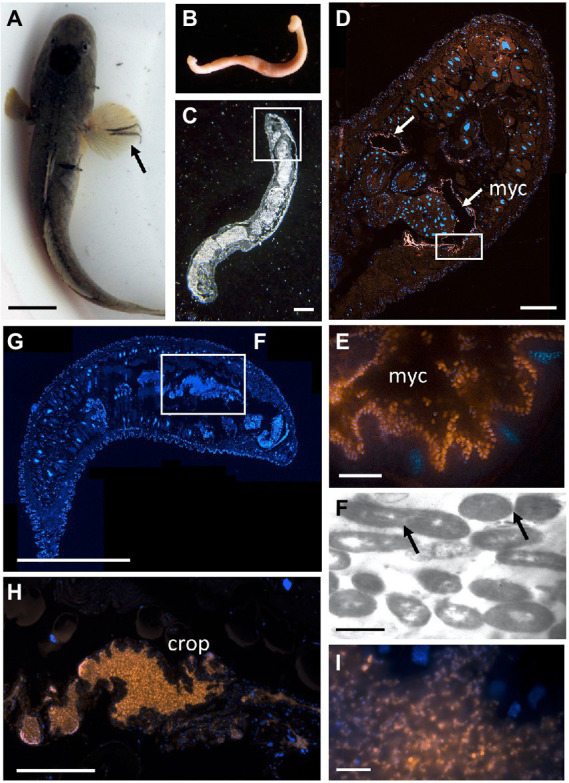
The bony fish leech *Pterobdella occidentalis* **(A)** on the fins and body of the longjaw mudsucker (*Gillichthys mirabilis*), indicated by the arrow. Scale bar 1 cm. **(B)** Whole specimen, taken with a Pentax WG-III handheld camera. **(C)** Specimen embedded in Steedman’s wax and sectioned, imaged *via* light microscopy. Square indicates region in **D**. Scale bar, 1 mm. **(D)** Longitudinal section through the anterior end, showing general bacteria cells in a pair of mycetomes (shown in orange, *via* Eub338-Cy3), counterstained with DAPI, shown in blue. Square indicates region in **E**. Scale bar, 200 μm. **(E)** Close-up of bacterial cells, shown in orange, attached to the putative mycetome epithelia. Scale bar is 10 μm. **(F)** Transmission electron microscopy of a putative mycetome, revealing bacteria-like cells, some which appear to be dividing (arrows). Scale bar, 1 μm. **(G)** Longitudinal section through a near complete leech specimen, stained with DAPI. Scale bar, 1 mm. **(H)** *Vibrio*-specific cells within the crop (shown in orange, *via* Cy3), counterstained with DAPI, shown in blue. Scale bar, 200 μm. **(I)**. Close-up of bacterial cells, shown in orange, within the crop. Scale bar, 10 μm. myc, mycetomes.

Since *Vibrio* was the most common bacterial group observed *via* 16S rRNA gene barcoding and cloning of OBF marine taxa, the ultrastructural position of *Vibrio* cells within specific tissues was examined (Figures 4–7; Supplementary Figure S4). Using specific fluorescence *in situ* hybridization microscopy, *Vibrio* cells were clearly observed within the lumen of the copepod *Lernanthropus*, among partially-digested bloodmeal (Figure 4). The morphology of siphonostomid copepods varies considerably depending on the precise attachment to their host fish (e.g., holdfast structures and abdominal lobes; Boxshall, 1986), thus we might expect to see a varied integration with *Vibrio*, or other bacterial associates, depending on the species examined in the future. For *Elthusa Vibrio* cells were observed in the intestine lumen, in an area that appeared to be filled with blood based on the Wright stain, near a junction with the digestive ceca (Figures 5D,F). Note that during dissection, the entire *Elthusa* intestine was pulsing with blood, which evacuated through the mouth when pressure was applied. For the leeches *Branchellion* and *Pterobdella*, *Vibrio* cells were observed in large regions of the blood-filled crop, often in and among the obvious densely packed erythrocytes of the prey host (e.g., Figure 6D). From embedded *Branchellion* tissues, preliminary laser capture microdissection of these bacteria-containing regions, resulted in amplification of only *Vibrio* 16S rRNA gene from the captured tissue (Supplementary Figure S1). Similarly, crop tissues specifically excised from 5 *Pterobdella* specimens only yielded *Vibrio* 16S rRNA genes. The crop is the largest component of the leech digestive tract that stores the bloodmeal after ingestion. It is also the location of the symbionts detected in previous studies on hirudinid leeches (Graf et al., 2006; Worthen et al., 2006). Symbionts can be free in the lumen, like individual *Aeromonas* cells inside the medicinal leech crop, or attached as a biofilm to tissue epithelia, as in *Mucinivorans hirudinis* (Kikuchi and Graf, 2007; Nelson et al., 2015). Notably, a biofilm of bacteria, not identified as *Vibrio*, was observed in *Pterobdella* attached to the luminal epithelia of the unique paired mycetomes (or esophageal diverticula), possessed by this genus (Figure 7). Transmission electron (TEM) microscopy of these putative mycetomes revealed numerous bacteria-like cells, some dividing (Figure 7G). Mycetomes were excised from two *Pterobdella* specimens, and analyzed *via* direct 16S rRNA gene sequencing, revealing the singular presence the Oceanospirillaceae, suggesting that these bacteria reside in a location distinct from the co-occurring *Vibrio*. At least 3 leech genera within the family Glossiphoniidae possess diverse bacterial symbionts in esophageal organs (Kikuchi and Fukatsu, 2002; Perkins et al., 2005). It is novel, however, to observe a mycetome-associated symbiont in a member of the Piscicolidae (within the order Aryhnchobdellida; Graf et al., 2006). Bacteria have also been observed residing within nephridia and bladders of leech excretory systems (Graf et al., 2006; Kikuchi et al., 2009), however it is not currently known whether this occurs in marine OBF leeches as well. Finally, *Vibrio* cells within the digestive tissues in the marine OBF taxa were not only observed within the crop lumen, but also in association with what appeared to be hemocytes of the invertebrate parasite. For the isopod *Elthusa*, *Vibrio* cells were observed inside of cells, at a specific junction between the midgut and the anterior digestive ceca (Figure 5). Similarly, for *Branchellion*, *Vibrio* were not only among erythrocytes, but also intracellular in what appeared to be hemocytes given the obvious presence of multiple nuclei (Figures 6E,F). Hemocytes, which are invertebrate immune cells with a phagocytic function, are thought to play a major role in the establishment and maintenance of beneficial associations between bacteria and invertebrates. For example, in both the medicinal leech and the Hawaiian bobtail squid, hemocytes have been observed interacting with the resident bacterial community (Silver et al., 2007; McAnulty and Nyholm, 2016), suggesting that symbionts can modulate the cellular immune response of the host, and vice versa.

Beneficial bacteria are most often housed (or rather colonize and persist) in a particular tissue, and their position within the body of the host animal can give clues as to their particular role, if any. The position of *Vibrio* in and among the bloodmeal hints at a possible role in red blood cell digestion in the marine OBF examined in this study. *Vibrio* isolates cultivated from the tissues of *Branchellion*, *Pterobdella*, *Elthusa*, and *Lernanthropus* matched the most dominant 16S rRNA gene amplicons (Supplementary Figure S1). For all but one of these *Vibrio* isolates, an ability to effectively lyse vertebrate blood cells embedded in agar media was observed, providing evidence for this capability. Since red blood cells constitute the largest cellular component of blood, their efficient lysis is a central requirement for blood-feeding parasites. A similar ability to lyse erythrocytes, and subsequently establish in the leech gut, has been observed in *Aeromonas veronii*, the primary symbiont of *Hirudo verbana* (Maltz and Graf, 2011). Bacteria generally found in association with medicinal leeches are thought to play an important role in the specific digestion of the blood meal, as well as the detoxification of heme (Toh et al., 2010). The latter appears to be a significant, and possibly underestimated, obstacle for blood-feeding animals, given that heme can generate hydroxyl radicals and reactive oxygen species, capable of damaging proteins, lipids and DNA (Aft and Mueller, 1984). Other than initiating the digestion of erythrocytes, several other functions for the dominant crop bacteria have been proposed for hirudinid leeches, including providing essential nutrients to the host, prevention of other bacteria from colonizing the crop, and immunological priming of the host invertebrate (Graf, 2002; Maltz and Graf, 2011; Rio et al., 2016; Husnik, 2018). It is not yet known whether bacterial residents play an important role in overcoming the dietary hurdles of low digestibility and vitamin B deficiency in marine OBF taxa.

## Conclusion

While most blood-feeding animals examined so far host internal bacterial symbionts that aid in some aspect of their nutrition, nearly all studies have focused on terrestrial blood-feeders. In this study, persistent internal associations with bacteria were observed for 6 phylogenetically-diverse species of marine obligate blood-feeders, including leeches, isopods, and copepods. These blood-feeding invertebrates possessed microbiomes of low diversity, mostly dominated by only a few bacterial groups, that were significantly distinct from non-blood-feeding relatives, biological surfaces, and seawater. Notably, *Vibrio* was abundant in all individuals examined and microscopy revealed their localization to the blood-filled lumen spaces. This hints at a possible evolutionary convergence of this bacterial genus as an essential abettor for a diet based solely on blood from marine vertebrates. We have preliminary indications of *Vibrio* presence in other hematophagous copepods, as well, including *Haemobaphes* and *Kroyeria* species, and it will be interesting to examine other marine OBF phyla, for example nematodes and flatworms, to see whether this prevalence extends even further across the parasite tree of life. Stable bacterial communities over 3–5 years of collection for each group examined in this study suggests intimate and non-random bacterial associations. Further investigations will require additional specimens and time points to understand the pervasiveness of the putative partnerships, and the specificity or degree of taxonomic diversity among possible bacterial partners, and additional life stages to uncover possible strategies for bacterial perpetuation. Eggs taken from the crustaceans *Elthusa* and *Lernanthropus* had very similar microbial communities to the adults, suggesting possible transmission of bacteria from parent to offspring in some hematophagous taxa. The role of the microbes associated with marine blood-feeding invertebrates is not yet determined, but there is every reason to believe that these animals face the same challenges with their exclusive subsistence on vertebrate blood. The distinctiveness of their bacteria from those associated with the environment and other non-hematophagous animals, further suggests a specific, exclusive, and likely functional relationship.

## Data availability statement

The datasets presented in this study can be found in online repositories. The names of the repository/repositories and accession number(s) can be found at: Small Read Archive (PRJNA910167), GenBank (OP981048-OP981071), and https://doi.org/10.5061/dryad.vmcvdncx9.

## Ethics statement

Fish and elasmobranchs were collected from the coastal waters of southern California, either by hook and line, beach seine, fish trap or *via* trawl onboard expeditions in collaboration with the Orange County Sanitation District (OCSD)—Environmental Laboratory and Ocean Monitoring team. In all cases, permits to collect the fish hosts, from which we removed invertebrate parasites and, in most cases released, were held by RA (SC-13105 and S-190710005-22077-001) and SG (SC-10578).

## Author contributions

SG conceived the study, designed the research, performed molecular analyses, and wrote the paper, with contributions from SG, RA, RH, and JR. RA arranged collection of all samples and aided in our understanding of parasitic taxa. RH and JR performed the research, including amplicon sequencing analyses and fluorescence microscopy in the laboratory. All authors contributed to the article and approved the submitted version.

## Funding

Funding for this project was made possible by generous donations by Ron and Susan Hahn and a National Science Foundation grant to SG (IOS-1947309).

## Conflict of interest

The authors declare that the research was conducted in the absence of any commercial or financial relationships that could be construed as a potential conflict of interest.

## Publisher’s note

All claims expressed in this article are solely those of the authors and do not necessarily represent those of their affiliated organizations, or those of the publisher, the editors and the reviewers. Any product that may be evaluated in this article, or claim that may be made by its manufacturer, is not guaranteed or endorsed by the publisher.

## Supplementary material

SUPPLEMENTARY TABLE S1 Specimens analyzed in this study, showing specimen ID, collection year, location, and host vertebrate, listed in the same order as in the stacked bar charts in Figures 2E,F.

SUPPLEMENTARY TABLE S2 Results from pairwise one-way Analysis of Similarities (ANOSIM) analysis for all comparisons mentioned in the text.

SUPPLEMENTARY TABLE S3 Microbial taxa that contribute up to 70% of the community structure of the microbiome (based on 16S rRNA genes amplicons) for obligate blood feeding (OBF) leeches and crustaceans, and comparison environmental samples. Highlighted in green are the most common bacterial groups in the OBF taxa. Those highlighted in gray are the most common bacterial groups in the comparison samples (non-blood feeding relatives, biological surfaces, and seawater). As well as the Shannon index calculated based on bacterial 16S rRNA gene sequences recovered from barcoding.

SUPPLEMENTARY TABLE S4 A complete color key for Figures 2E,F, showing the relative abundance of bacterial community structure at the genus level, for marine blood-feeders examined in this study.

SUPPLEMENTARY FIGURE S1 Phylogenetic relationships among *Vibrio* associated with blood feeding marine crustaceans and leeches, relative to selected cultured representatives, based on the 16S rRNA gene. Sequences generated in this study are from clone libraries (“clone”), bacterial cultivation (“isolate”), and amplicon sequencing (“barcode”). A neighbor-joining tree with Jukes-Cantor two-parameter distances is shown. None of the nodes are supported by bootstraps, reinforcing the very close relationship among known *Vibrio* species, and the need for a more specific genomic taxonomy framework. Genbank accession numbers are indicated in parentheses. *Vibrio* barcodes shown in Figures 3 are highlighted in bold text. At right are scanning electron microscopy or blood agar hydrolysis images of bacterial isolates corresponding to the main *Vibrio* groups recovered from blood feeding marine crustaceans and leeches.

SUPPLEMENTARY FIGURE S2 Phylogenetic relationships of bacteria associated with blood feeding marine crustaceans and leeches (based on 16S rRNA gene amplicon sequencing, designated by names beginning with ‘OBF’), compared to select close relatives recovered during other investigations. Genbank accession numbers are indicated in parentheses. **(A)** gammaproteobacteria, including unclassified members and Porticoccaceae + Oceanospirillaceae, **(B)** alphaproteobacteria, including unclassified members and Rhodobacteraceae **(C)** betaproteobacteria, including Rhodocyclaceae, and **(D)** Flavobacteriacea. For each, a neighbor-joining tree with Jukes-Cantor two-parameter distances is shown. The numbers at the nodes represent maximum parsimony bootstrap values from 1000 replicate samplings (only values >60% are shown).

SUPPLEMENTARY FIGURE S3 Comparisons of the microbiome diversity of adult obligate blood feeding crustaceans and leeches, versus egg and cocoons, respectively. 16S rRNA gene sequence similarity was analyzed using a non-metric multidimensional scaling (NMDS) ordination plot based on Bray-Curtis similarity resemblance. The tables below compare the Shannon diversity index in both the adults and eggs (or cocoons), as well as the relative abundance of select dominant bacterial groups.

SUPPLEMENTARY FIGURE S4 Fluorescent visualization using a nonsense probe, labeled with a Cy3 fluorescent molecule, counterstained with DAPI. **(A)** The bony fish leech *Pterobdella occidentalis*, showing a negative signal compared to the positive signal shown in Figures 7H. Scale bar 200 μm. **(B)** The parasitic isopod *Elthusa vulgaris*, showing a negative signal compared to the positive signal shown in Figures 7F. Scale bar 100 μm. **(C)** *Lernanthropus latis,* showing a negative signal, compared to panel E. Scale bar 20 μm. **(D)** The elasmobranch leech *Branchellion lobata*, showing a negative signal, compared to panel F. Scale bar 20 μm. **(E)** *Lernanthropus latis,* showing the result from the *Vibrio*-specific fluorescent probe revealing rod-shaped bacterial cells, shown in orange via Cy3. Scale bar 20 μm. (F) *Branchellion lobata,* showing the result from the *Vibrio*-specific fluorescent probe revealing rod-shaped bacterial cells, shown in orange via Cy3. Scale bar 20 μm.
