# Supplementary material for: Marine vampires: Persistent, internal associations between bacteria and blood-feeding marine annelids and crustaceans
Source: Front Microbiol. 2023 Jan 11;13:1113237. doi: 10.3389/fmicb.2022.1113237 (PMC9876621; doi:10.3389/fmicb.2022.1113237)
Supplement: SUPPLEMENTARY TABLE S1 — Specimens analyzed in this study, showing specimen ID, collection year, location, and host vertebrate, listed in the same order as in the stacked bar charts in Figures 2E,F. [file Data_Sheet_1.PDF]

Table S1

Marine parasite species	Sample ID	Collection Location	Year	Host	barcode label (SRA files)
Elthusa vulgaris	SGCs150730	OCSD station 11	2015	*Citharichthys sordidus*	Elthusa-1_S3
	SGCs150730	OCSD station 11	2015	*Citharichthys sordidus*	Elthusa-2_S1
	SGCs150730	OCSD station 11	2015	*Citharichthys sordidus*	Elthusa-3_S2
	SGXx160727	OCSD station 11	2016	*unknown*	Elthusa-F_S248
	SGXx160727	OCSD station 11	2016	*unknown*	Elthusa-G_S27
	SGXx160727	OCSD station 11	2016	*unknown*	Elthusa-H_S28
	SGXx160727	OCSD station 11	2016	*unknown*	Elthusa-I_S29
	SGXx160727	OCSD station 11	2016	*unknown*	Elthusa-J_S30
	SGXx160727	OCSD station 11	2016	*unknown*	Elthusa-L_S222
	SGXx160727	OCSD station 11	2016	*unknown*	Elthusa-M_S223
	SGXx160727	OCSD station 11	2016	*unknown*	Elthusa-O_S224
	SGXx161105	Palos Verdes Peninsula	2016	*unknown*	Elthusa-G1-1gut_S227
	RAFp180117-9	Anahem Bay/National Wildlife Refuge	2018	*Fundulus parvipinnis*	Elthusa-killifish-G1_S5
	RAFp180117-9	Anahem Bay/National Wildlife Refuge	2018	*Fundulus parvipinnis*	Elthusa-killifish-G2_S6
	RAFp180117-9	Anahem Bay/National Wildlife Refuge	2018	*Fundulus parvipinnis*	Elthusa-killifish-G3_S7
	SGXx160727	eggs	2016	*unknown*	Elthusa-I-eggs_S245
	SGXx161105	eggs	2016	*unknown*	Elthusa-G1-1eggs_S244
	SGCs150730	eggs	2016	*unknown*	Elthusa-3-eggs_S249
Nerocila californica	RACa150412-2	San Diego Bay	2015	*Cymatogaster aggregata*	Nerocila-RACa150412-2_S167
	RAPc160714-4	San Diego Bay	2016	*Parlabrax clathratus*	Nerocila-RAPc160714-4_S165
Lenanthropus latis	RAMu161027-1	El Segundo Beach, CA	2016	*Menticirrus undulatus*	LenanthropusA_S233
	Mu200604	Redondo Beach, CA	2020	*Menticirrus undulatus*	Lerna-Mu200604_S237
	Mu200715	El Segundo Beach, CA	2020	*Menticirrus undulatus*	Lerna-Mu200715_S238
	RaMu190917-2	Redondo Beach, CA	2019	*Menticirrus undulatus*	Lerna-RaMu190917-2_S210
	RACns210806.2	Mission Bay	2021	*Cheilotrema saturnum*	Lerna-RACns210806-2_S217
	RAMu160303-1	Inner Cabrillo Beach	2016	*Menticirrus undulatus*	Lerna-W5A_S35
	RAMu170707-1	El Segundo Beach, CA	2017	*Menticirrus undulatus*	Lerna-W9A_S25
	RAMu170707-1	El Segundo Beach, CA	2017	*Menticirrus undulatus*	Lerna-W9B_S20
	RAMu170707-1	eggs	2017	*Menticirrus undulatus*	Lerna-eggs-RaMu-W9A_S224
	RAMu160303-1	eggs	2016	*Menticirrus undulatus*	Lerna-eggs-RaMu-W5A_S225
	RaMu190917-2	eggs	2019	*Menticirrus undulatus*	Lerna-eggs-RaMu190917-2_S223
Non-blood feeding copepod	RANc180211-1	Mission Bay	2018	*Neotrypaea californiensis*	NBF-copepod-RANc180211-1_S18
	RANc180211-1	Mission Bay	2018	*Neotrypaea californiensis*	NBF-copepod2-RANc180211-1_S19
	SG150915-1	LA Harbor	2015	*Skate*	NBF-copepod-SG150915-1_S11
	RAUh180212-2	San Diego Bay	2018	*Urobatis halleri*	NBF-copepod-RAUh180212-2_S17
Ghost shrimp exoskeleton	RANc180211-1	Mission Bay	2018	*Neotrypaea californiensis*	Shrimp_gillcover2-RANc180211-1_S34
Non-blood feeding copepod	SGUh201205-1	LA Harbor	2020	*Urobatis halleri*	NBF-copepod-sealice_S83
	RARp180418-2	Seal Beach	2018	*Rhinobatus productus*	NBF-sealouse_RARp180418-2
Ghost shrimp exoskeleton	RANc180211-1	Mission Bay	2018	*Neotrypaea californiensis*	Shrimp_gillcover-RANc180211-1_S33
Seawater	seawater-SWP	LA Harbor	2015	*N/A*	seawater-SWP_S256
	seawater-SWB	Anaheim Bay, CA	2015	*N/A*	seawater-SWB_S255
	seawater-WOA	Palos Verdes	2015	*N/A*	seawater-WOA_S248
Branchellion lobata	RAUh161101-1	LA Harbor	2016	*Urobatis halleri*	Branch-W9B_S250
	RAGma150721.5	San Diego Bay	2015	*Gymnura marmorata*	Branch-RAGma150721-5_S209
	RAMc150411.1	San Diego Bay	2015	*Myliobatis californica*	Branch-RAMc150411-1_S210
	RARp180418.2	Seal Beach	2018	*Rhinobatus productus*	Branch-RARp180418-2_S208
	RAUh180129-1	South San Diego Bay	2018	*Urobatis halleri*	Branch-RAUh180129-1_S212
	RAUh190708-2	North San Diego Bay	2019	*Urobatis halleri*	Branch-RAUh190708-2_S213
	RAMyc180720-3b	Colorado Lagoon, Long Beach	2018	*Myliobatis californica*	Branch-BlK-RAMyc180720-3b_S81
	RAUh170808-1	LA Harbor	2017	*Urobatis halleri*	Branch-T1A_S26
	RAUh170808-1	LA Harbor	2017	*Urobatis halleri*	Branch-T1B_S15
	RAUh170906-1	Anaheim Bay	2017	*Urobatis halleri*	Branch-W6A_S23
	RAUh170906-1	Anaheim Bay	2017	*Urobatis halleri*	Branch-W6B_S14
	RAMyc180720-3a	Colorado Lagoon, Long Beach	2018	*Myliobatis californica*	Branch-Wht-RAMyc180720-3a_S79
Ostreobdella californiana	FDSxSA2017	Steinhart Aquarium	2017	*Sebastes spp.*	Ostreobdella-FD1_S220
	FDSxSA2017	Steinhart Aquarium	2017	*Sebastes spp.*	Ostreobdella-FD2_S251
	FDSxSA2017	Steinhart Aquarium	2017	*Sebastes spp.*	Ostreobdella-PC1_S154
	FDSxSA2017	Steinhart Aquarium	2017	*Sebastes spp.*	Ostreobdella-PC2_S155
	FDSxSA2017	Steinhart Aquarium	2017	*Sebastes spp.*	Ostreobdella-SG1_S156
	FDSxSA2017	Steinhart Aquarium	2017	*Sebastes spp.*	Ostreobdella-SG2_S157
	FDSxSA2017	Steinhart Aquarium	2017	*Sebastes spp.*	Ostreobdella-SG3_S160
Pterobdella occidentalis	RAGm210604-2	Anaheim Bay, CA	2021	*Gillichthys mirabilis*	Pterob-2_S208
	RAGm210604-2	Anaheim Bay, CA	2021	*Gillichthys mirabilis*	Pterob-1_S230
	RAGm160805-1	Bolsa, Pocket Marsh	2016	*Gillichthys mirabilis*	Pterob-adult1_S12
	RAGm160805-1	Bolsa, Pocket Marsh	2016	*Gillichthys mirabilis*	Pterob-adult1b_S20
	RAGm160805-1	Bolsa, Pocket Marsh	2016	*Gillichthys mirabilis*	Pterob-adult2_S13
	RAGm160805-1	Bolsa, Pocket Marsh	2016	*Gillichthys mirabilis*	Pterob-adult2b_S21
	RAGm160805-1	Bolsa, Pocket Marsh	2016	*Gillichthys mirabilis*	Pterob-adult3b_S22
	RAGm160805-1	Bolsa, Pocket Marsh	2016	*Gillichthys mirabilis*	Pterob-juv1_S10
	RAGm160805-1	Bolsa, Pocket Marsh	2016	*Gillichthys mirabilis*	Pterob-juv1b_S18

	RAGm160805-1	Bolsa, Pocket Marsh	2016	*Gillichthys mirabilis*	Pterob-juv2_S11
	RAGm160805-1	Bolsa, Pocket Marsh	2016	*Gillichthys mirabilis*	Pterob-juv2b_S19
	RAGm190802-5	Morro Bay	2019	*Gillichthys mirabilis*	Pterob-RAGm190802_5Nov
	RAGm190802-17	Morro Bay	2019	*Gillichthys mirabilis*	Pterob-RAGm190802_17Nov
	RALa200805.C.1	Willapa Bay, OR	2020	*Leptocottus armatus*	Pterob-RALa200805-C-1_S232
	.RALa200805.C.2	Willapa Bay, OR	2020	*Leptocottus armatus*	Pterob-RALa200805-C-2_S233
	RALa200805.C.3	Willapa Bay, OR	2020	*Leptocottus armatus*	Pterob-RALa200805-C-3_S234
	RAAf180926-1-lg	Upper Newport Bay	2018	*Acanthogobius flavimanus*	Pterob-goby-1_S11
	RAAf180926-1-sm	Upper Newport Bay	2018	*Acanthogobius flavimanus*	Pterob-goby-2_S12
	RAGm160805-1	cocoons	2016	*N/A*	PterobCocoons_S9
	RAGm160805-1	cocoons	2016	*N/A*	PterobCocoons-b_S17
	RAPc211203.3	cocoons	2021	*N/A*	PterobCocoons-RAPc211203-3_S203
Pterobdella abditovesiculata	RAEs220204.1	Wailoa River estuary, HI	2022	*Eleotris sandwicensis*	Pterob-RAEs220204-1-Hawaii4_S205

Swabs from fish skin	PCGm180124-1	Anaheim Bay	2018	*Gillichthys mirabilis*	Fish-Swab-PCGm180124-1_S1
	PCGm180124-2	Anaheim Bay	2018	*Gillichthys mirabilis*	Fish-Swab-PCGm180124-2_S2
	PCGm180124-3	Anaheim Bay	2018	*Gillichthys mirabilis*	Fish-Swab-PCGm180124-3_S3
	PCGm180124-4	Anaheim Bay	2018	*Gillichthys mirabilis*	Fish-Swab-PCGm180124-4_S4
	RAGm180207-1	Anaheim Bay	2018	*Gillichthys mirabilis*	Fish-Swab-Gm28-5-20_S195
	RAGm180129-9	Sweetwater Marsh, San Diego Bay	2018	*Gillichthys mirabilis*	Fish-Swab-Gm52-6-12_S196
	RAGm180129-9	Sweetwater Marsh, San Diego Bay	2018	*Gillichthys mirabilis*	Fish-Swab-Gm52-6-23_S194
	RAGm180129-4	Sweetwater Marsh, San Diego Bay	2018	*Gillichthys mirabilis*	Fish-Swab-Gm52-7-6b_S197
	RAFp180207-1	Anaheim Bay	2018	*Fundulus parvipinnis*	Fish-Swab-RAFp180207-1_S192

Table S2

Analysis of Similarities -- One-Way ANOSIM

**Pairwise Tests**<br>**Groups**	**R**<br>**Statistic**	**Significance**<br>**Level %**	**Conclusion**
OBF_Crustacean vs. NonBF_Crustacean	0.548	0.1	different
OBF_Crustacean vs. Seawater	0.870	0.1	different
NonBF_Crustacean vs. Seawater	0.454	3.6	different
OBF_leech vs. Swabs	0.433	0.1	different
OBF_leech vs. Seawater	0.652	0.1	different
Swabs vs. Seawater	0.839	0.3	different
Lernanthropus vs. Elthusa	0.528	0.1	different
Nerocila vs. Elthusa	0.558	0.7	different
Lernanthropus vs. Nerocila	0.909	2.2	different
Branchellion vs. Pterobdella	0.648	0.1	different
Branchellion vs. Ostreobdella	0.999	0.1	different
Pterobdella vs. Ostreobdella	0.923	0.1	different
Elthusa eggs vs. adults	0.225	14.1	not different
Lernantrhopus eggs vs. adults	-0.121	7.3	not different
Pterobdella cocoons vs. adults	0.987	0.3	different
Lernantrhopus by year	0.573	6.2	not different
Pterobdella by year (and location)	0.83	0.1	different
Branchellion by year	0.27	8.7	not different

Table S3: Microbial taxa (based on % of 16S rRNA genes) that contribute up to 70% of the community structure of the microbiome for obligate blood feeding leeches and crustaceans, and comparison environmental samples

	**Branchellion**		**Ostreobdella**		**Pterobdella**		**Elthusa**		**Lernanthropus**		**Nerocila**		**NonBF-Crustaceans**		**Swabs**		**Seawater**	
**Shannon Index**	1.45	0.58	1.00	0.32	1.49	0.56	1.36	0.64	1.75	0.4	0.48	0.61	2.23	0.4	2.5	0.4	1.87	0.43
**Taxa**	Av.Abund	SD	Av.Abund	SD	Av.Abund	SD	Av.Abund	SD	Av.Abund	SD	Av.Abund	SD	Av.Abund	SD	Av.Abund	SD	Av.Abund	SD
Vibrionaceae; Vibrio	51.2	6.8	69.6	4.8	40.2	6.8	67.4	8.2	54.4	5.5	61.5		23.4	2.4	35.7	2.4		
Vibrionaceae; Photobacterium									18.5	5.5								
Flavobacteriaceae			22.6	4.0			7.5	1.6					14.6	2.2	10.4	0.9	6.6	0.6
Thalassolitus_Marinobacterium					33.6	9.6											13.7	3.5
Shewanella							20.5	5.7										
Porticoccaceae									23.5	5.4								
Rhodocyclaceae	16.6	5.5																
Gammaproteobacteria	15.0	3.0							8.8	1.5			10.9	0.5				
Cellvibrionales	11.9	2.8																
Rhodobacteraceae							10.3	1.4					16.9	1.9	15.8	1.7	19.0	3.1
Cellvibrionaceae					8.3	2.1												
Alteromonas													15.3	3.8				
Alphaproteobacteria													13.9	3.1				
Pseudoalteromonas													12.1	2.2				
Enterovibrio													10.8	1.9				
Tenacibaculum													8.8	1.3	8.5	1.2		
Alteromonadaceae													6.6	1.4				
Thiotrichaceae													5.6	0.9				
Marinomonas															23.1	3.8		
Pseudoalteromonas															20.0	2.7		
Rubritalea															12.1	1.5		
Gammaproteobacteria2															8.2	0.6		
Neptuniibacter															8.9	1.1		
Crocinitomicaceae															7.8	0.9		
Colwelliaceae															4.8	0.5		
Cand_Nitrosopumilus																	12.0	0.5
Cryomorphaceae																	13.2	3.8
NS9_marine_group																	6.1	1.3
Unassigned;Otherlesspt1pct	10.3	1.2	10.7	0.8	8.1	1.0	9.8	1.5	13.9	1.4	x	x	18.7	1.1	19.8	0.8	41.0	1.5

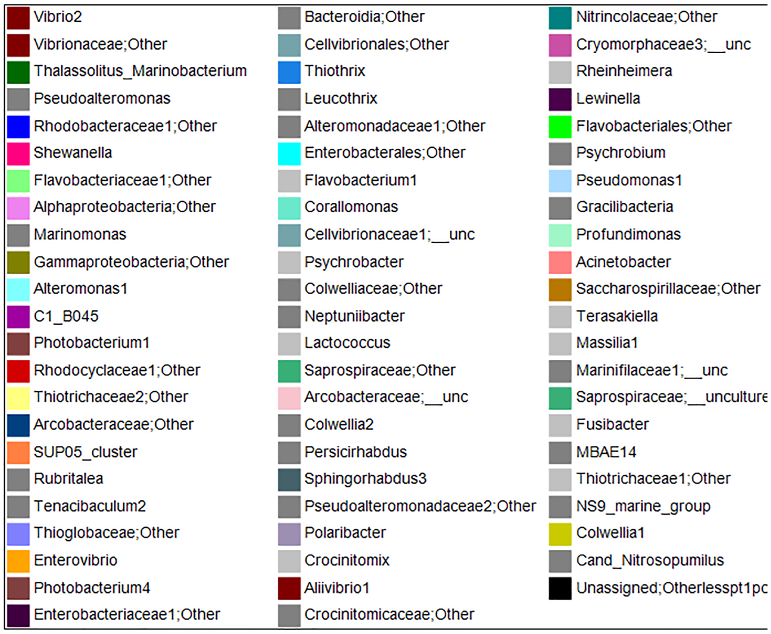
Vibrio2
Vibrionaceae;Other
Thalassolitus_Marinobacterium
Pseudoalteromonas
Rhodobacteraceae1;Other
Shewanella
Flavobacteriaceae1;Other
Alphaproteobacteria;Other
Marinomonas
Gammaproteobacteria;Other
Alteromonas1
C1_B045
Photobacterium1
Rhodocyclaceae1;Other
Thiotrichaceae2;Other
Arcobacteraceae;Other
SUP05_cluster
Rubritalea
Tenacibaculum2
Thioglobaceae;Other
Enterovibrio
Photobacterium4
Enterobacteriaceae1;Other
Bacteroidia;Other
Cellvibrionales;Other
Thiothrix
Leucothrix
Alteromonadaceae1;Other
Enterobacterales;Other
Flavobacterium1
Corallomonas
Cellvibrionaceae1;__unc
Psychrobacter
Colwelliaceae;Other
Neptuniibacter
Lactococcus
Saprospiraceae;Other
Arcobacteraceae;__unc
Colwellia2
Persicirhabdus
Sphingorhabdus3
Pseudoalteromonadaceae2;Other
Polaribacter
Crocinitomix
Aliivibrio1
Crocinitomicaceae;Other
Nitrincolaceae;Other
Cryomorphaceae3;__unc
Rheinheimera
Lewinella
Flavobacteriales;Other
Psychrobium
Pseudomonas1
Gracilibacteria
Profundimonas
Acinetobacter
Saccharospirillaceae;Other
Terasakiella
Massilia1
Marinifilaceae1;__unc
Saprospiraceae;__uncultur
Fusibacter
MBAE14
Thiotrichaceae1;Other
NS9_marine_group
Colwellia1
Cand_Nitrosopumilus
Unassigned;Otherlesspt1pc

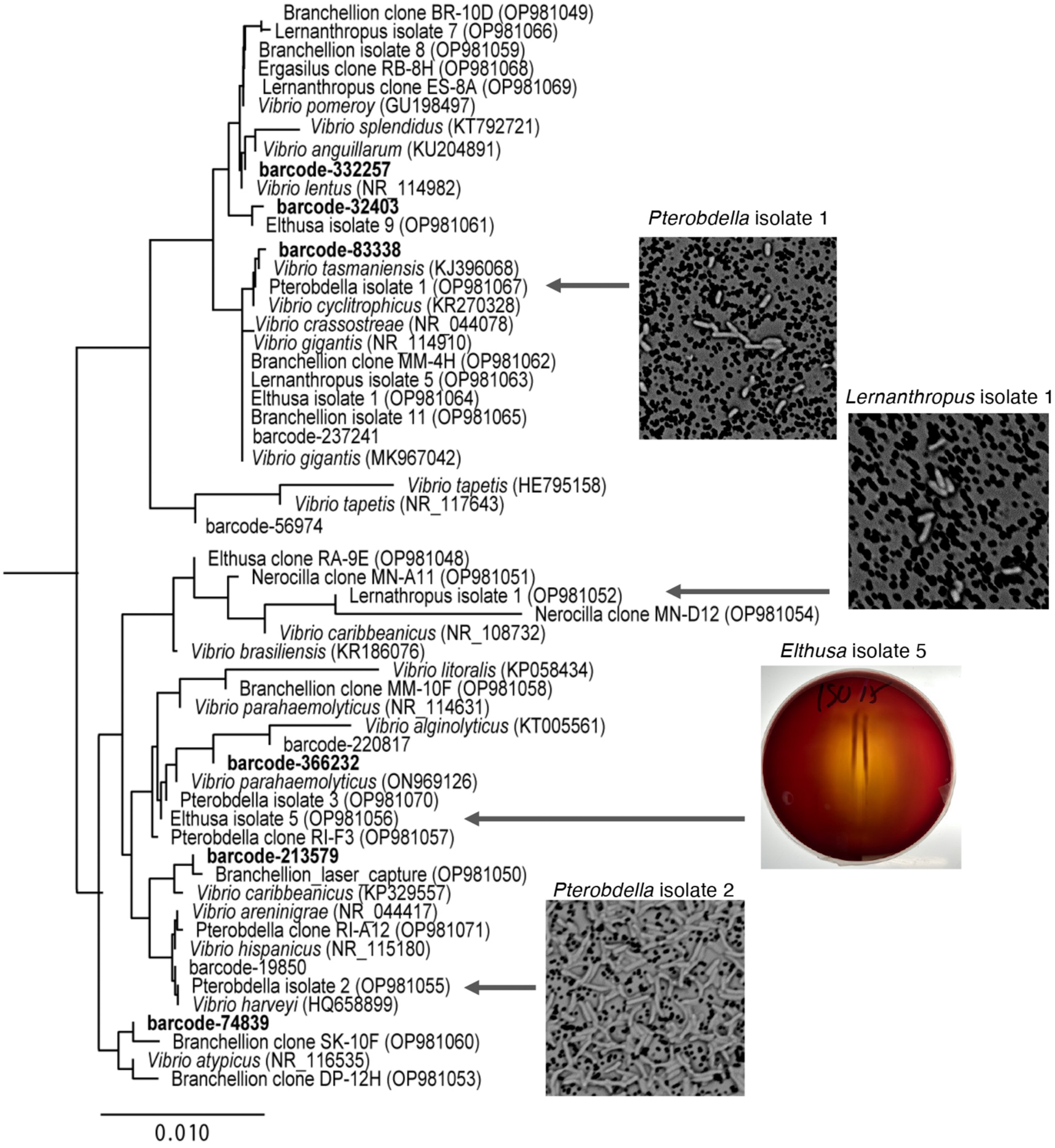
Branchellion clone BR-10D (OP981049)
Lernanthropus isolate 7 (OP981066)
Branchellion isolate 8 (OP981059)
Ergasilus clone RB-8H (OP981068)
Lernanthropus clone ES-8A (OP981069)
Vibrio pomeroy (GU198497)
Vibrio splendidus (KT792721)
Vibrio anguillarum (KU204891)
barcode-332257
Vibrio lentus (NR_114982)
barcode-32403
Elthusa isolate 9 (OP981061)
barcode-83338
Vibrio tasmaniensis (KJ396068)
Pterobdella isolate 1 (OP981067)
Vibrio cyclitrophicus (KR270328)
Vibrio crassostreae (NR_044078)
Vibrio gigantis (NR_114910)
Branchellion clone MM-4H (OP981062)
Lernanthropus isolate 5 (OP981063)
Elthusa isolate 1 (OP981064)
Branchellion isolate 11 (OP981065)
barcode-237241
Vibrio gigantis (MK967042)
Vibrio tapetis (HE795158)
Vibrio tapetis (NR_117643)
barcode-56974
Elthusa clone RA-9E (OP981048)
Nerocilla clone MN-A11 (OP981051)
Lernathropus isolate 1 (OP981052)
Nerocilla clone MN-D12 (OP981054)
Vibrio caribbeanicus (NR_108732)
Vibrio brasiliensis (KR186076)
Vibrio litoralis (KP058434)
Branchellion clone MM-10F (OP981058)
Vibrio parahaemolyticus (NR_114631)
Vibrio alginolyticus (KT005561)
barcode-220817
barcode-366232
Vibrio parahaemolyticus (ON969126)
Pterobdella isolate 3 (OP981070)
Elthusa isolate 5 (OP981056)
Pterobdella clone RI-F3 (OP981057)
barcode-213579
Branchellion_laser_capture (OP981050)
Vibrio caribbeanicus (KP329557)
Vibrio areninigrae (NR_044417)
Pterobdella clone RI-A12 (OP981071)
Vibrio hispanicus (NR_115180)
barcode-19850
Pterobdella isolate 2 (OP981055)
Vibrio harveyi (HQ658899)
barcode-74839
Branchellion clone SK-10F (OP981060)
Vibrio atypicus (NR_116535)
Branchellion clone DP-12H (OP981053)
0.010
Pterobdella isolate 1
Lernanthropus isolate 1
Elthusa isolate 5
Pterobdella isolate 2

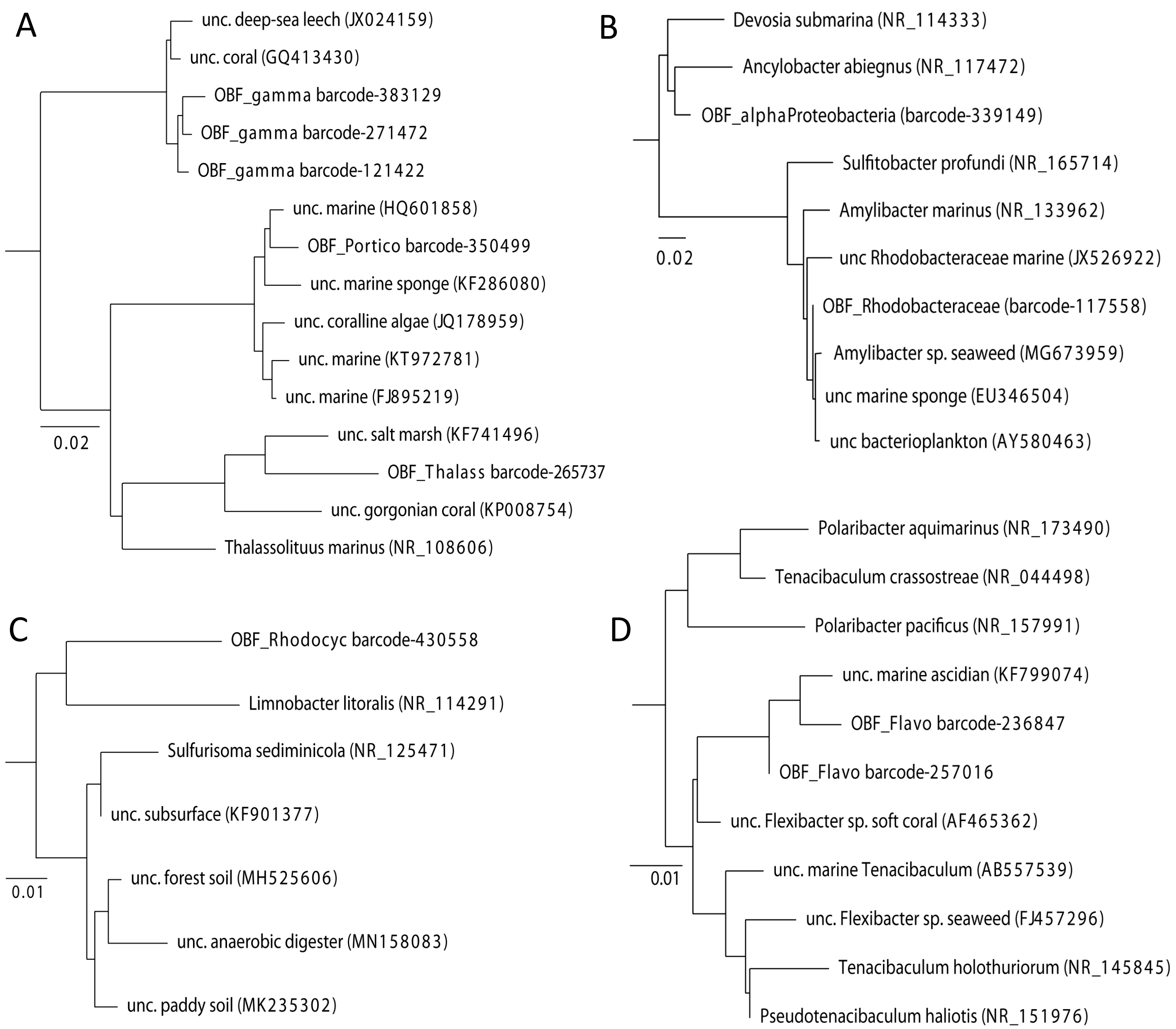
A
unc. deep-sea leech (JX024159)
unc. coral (GQ413430)
OBF_gamma barcode-383129
OBF_gamma barcode-271472
OBF_gamma barcode-121422
unc. marine (HQ601858)
OBF_Portico barcode-350499
unc. marine sponge (KF286080)
unc. coralline algae (JQ178959)
unc. marine (KT972781)
unc. marine (FJ895219)
0.02
unc. salt marsh (KF741496)
OBF_Thalass barcode-265737
unc. gorgonian coral (KP008754)
Thalassolituus marinus (NR_108606)
B
Devosia submarina (NR_114333)
Ancylobacter abiegnus (NR_117472)
OBF_alphaProteobacteria (barcode-339149)
Sulfitobacter profundi (NR_165714)
Amylibacter marinus (NR_133962)
0.02
unc Rhodobacteraceae marine (JX526922)
OBF_Rhodobacteraceae (barcode-117558)
Amylibacter sp. seaweed (MG673959)
unc marine sponge (EU346504)
unc bacterioplankton (AY580463)
C
OBF_Rhodocyc barcode-430558
Limnobacter litoralis (NR_114291)
Sulfurisoma sediminicola (NR_125471)
unc. subsurface (KF901377)
0.01
unc. forest soil (MH525606)
unc. anaerobic digester (MN158083)
unc. paddy soil (MK235302)
D
Polaribacter aquimarinus (NR_173490)
Tenacibaculum crassostreae (NR_044498)
Polaribacter pacificus (NR_157991)
unc. marine ascidian (KF799074)
OBF_Flavo barcode-236847
OBF_Flavo barcode-257016
unc. Flexibacter sp. soft coral (AF465362)
0.01
unc. marine Tenacibaculum (AB557539)
unc. Flexibacter sp. seaweed (FJ457296)
Tenacibaculum holothuriorum (NR_145845)
Pseudotenacibaculum haliotis (NR_151976)

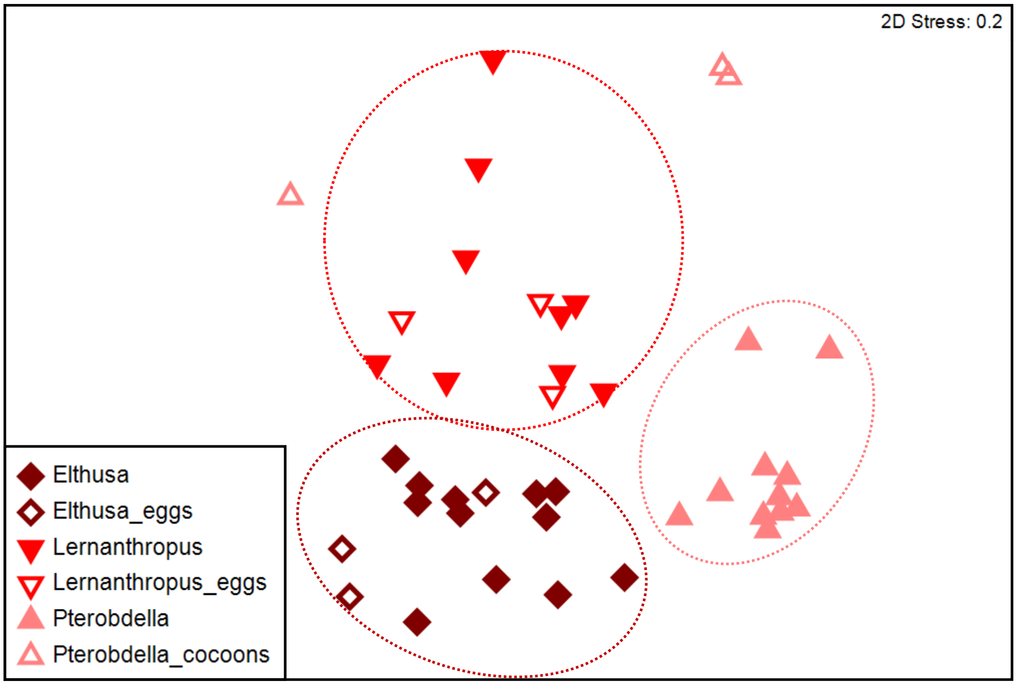

Elthusa	Adult	Eggs
Shannon Index	1.24	1.63
Vibrio	67.0	43.0
Shewanella	24.9	22.8
Pseudoalteromonas	8.6	41.2
Psychrobacter	0.6	14.2

Lernanthropus	Adult	Eggs
Shannon Index	1.79	1.90
Vibrio	49.12	58.19
Photobacterium	21.04	38.19
Porticoccaceae	26.67	18.1
Pseudoalteromonas	6.69	10.31

Pterobdella	Adult	Cocoons
Shannon Index	1.20	2.97
Vibrio	43.4	8.1
Oceanospirillaceae	42.7	5.8
Rhodobacteraceae	6.4	29.5
Nitrospira	1.1	14.0
Nitrosomonas	0.2	11.9
Saprospiraceae	1.8	11.6

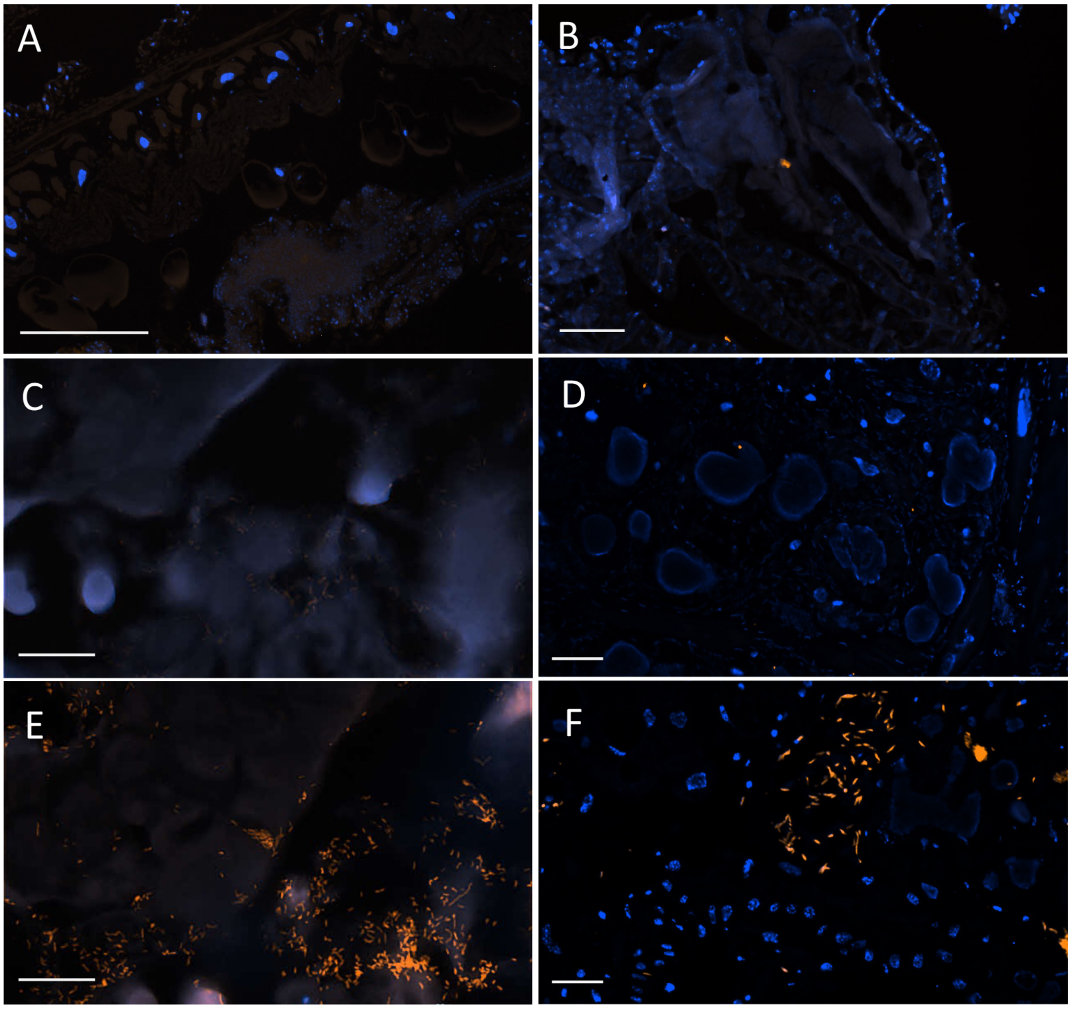
A
B
C
D
E
F
